# Differential assessment of skeletal, alveolar, and dental components induced by microimplant-supported midfacial skeletal expander (MSE), utilizing novel angular measurements from the fulcrum

**DOI:** 10.1186/s40510-020-00320-w

**Published:** 2020-07-13

**Authors:** Ney Paredes, Ozge Colak, Luca Sfogliano, Islam Elkenawy, Layla Fijany, Andrew Fraser, Boshi Zhang, Won Moon

**Affiliations:** Division of Growth and Development, Section of Orthodontics, School of Dentistry, Center for Health Science, University of California, Los Angeles, Room 63-082 CHS, 10833 Le Conte Ave, Box 951668, Los Angeles, CA 90095-1668 USA

**Keywords:** Cone-beam computed tomography (CBCT), Microimplant-assisted rapid palatal expansion (MARPE), Expansion, Alveolar bone bending, Dental tipping

## Abstract

**Background:**

In order to assess skeletal expansion, alveolar bone bending, and dental tipping after maxillary expansion, linear and angular measurements have been performed utilizing different craniofacial references. Since the expansion with midfacial skeletal expander (MSE) is archial in nature, the aim of this paper is to quantify the differential components of MSE expansion by calculating the fulcrum locations and applying a novel angular measurement system.

**Methods:**

Thirty-nine subjects with a mean age of 18.2 ± 4.2 years were treated with MSE. Pre- and post-expansion CBCT records were superimposed and compared. The rotational fulcrum of the zygomaticomaxillary complex was identified by localizing the interfrontal distance and modified interfrontal distance. Based on the fulcrum, a novel angular measurement method is presented and compared with a conventional linear method to assess changes of the zygomaticomaxillary complex, dentoalveolar bone, and maxillary first molars.

**Results:**

From 39 patients, 20 subjects have the rotational fulcrum of the zygomaticomaxillary complex at the most distant points of the interfrontal distance (101.6 ± 4.7 mm) and 19 subjects at the most distant points of the modified interfrontal distance (98.9 ± 5.7 mm). Linear measurements accounted for 60.16% and 56.83% of skeletal expansion, 16.15% and 16.55% of alveolar bone bending, and 23.69% and 26.62% of dental tipping for right and left side. Angular measurements showed 96.58% and 95.44% of skeletal expansion, 0.34% and 0.33% alveolar bone bending, and 3.08% and 4.23% of dental tipping for the right and left sides. The frontozygomatic, frontoalveolar, and frontodental angles were not significant different (P > 0.05).

**Conclusions:**

In the coronal plane, the center of rotation for the zygomaticomaxillary complex was located at the most external and inferior point of the zygomatic process of the frontal bone or slightly above and parallel to the interfrontal distance. Due to the rotational displacement of the zygomaticomaxillary complex, angular measurements should be a preferred method for assessing the expansion effects, instead of the traditional linear measurement method.

## Highlights

- In the coronal plane, the center of rotation for the zygomaticomaxillary complex with the midfacial skeletal expander (MSE), was located at the most external and inferior point of the zygomatic process of the frontal bone or slightly above and parallel to the interfrontal distance.
- Due to the rotational displacement of the zygomaticomaxillary complex, angular measurements should be the preferred method for accurately assessing the expansion effects, instead of traditional linear measurements.
- Traditional linear measurements underestimate orthopedic expansion effects and overestimate dentoalveolar side-effects.
- For each expander appliance, a precise location of fulcrum should be determined for accurate measurements.

## Introduction

Maxillary transverse deficiency is probably one of the most common skeletal problems in the craniofacial region. Fortunately, the transverse dimension of maxilla may be the most malleable of the craniofacial complex [1]. Rapid palatal expansion (RPE) has been the preferred standard treatment when a transverse deficit is present, especially in young patients. While the main goal of RPE is to split the midpalatal suture, the circum-maxillary sutures are also affected [2] and bone bending and dental tipping are common [3–6]. The desire is to produce a greater skeletal effect than dentoalveolar side-effects; however, the latter are commonly expressed in substantive magnitude.

When RPE treatment is performed before the pubertal growth spurt, the skeletal expansion predominates over the dentoalveolar changes [7]. However, a significant alveolar bone bending and dental tipping cannot be ignored, even in these young populations. Dentoalveolar changes are associated with a decrease in alveolar bone height, fenestration, and bone dehiscence [8]. These negative effects escalate in mature patients because of the difficulty in splitting the heavily interlocked midpalatal suture with tooth-borne appliance. In order to overcome the undesired dentoalveolar effects of RPE, a variety of bone-borne or hybrid microimplant-assisted rapid palatal expanders (MARPE) have been designed by many investigators [9–19]. In recent years, MARPE became popular in attempts to minimize the negative side-effects discussed above. These new breeds of expanders offered more bone anchor than the traditional tooth-borne RPE; however, the results varied significantly from one appliance to another because of the difference in appliance design and expansion protocols. Even with the bone anchor, significant dentoalveolar changes have been reported in many MARPE studies [11, 14–20]. While a paper reported a negligible molar tipping [21], others claimed that dental tipping and alveolar bone bending are not preventable but presented a different percentage of dentoalveolar changes between RPE and MARPE [9, 10].

Midfacial skeletal expander (MSE) is a particular type of MARPE which has been described in the literature since 2014 [12, 20–29]. The impacts of appliance has been thoroughly studied and described in the recent years, and it has been successfully applied in mature patients [12, 21, 28]. Cantarella illustrated that the zygomaticomaxillary complex, along with its inferior structures, move in a downward and outward direction in the coronal plane with a fulcrum localized slightly above the frontozygomatic suture [21].

In assessing percentages of skeletal, alveolar, and dental components after maxillary expansion with RPE and MARPE, various linear measurements and angular measurements from arbitrary points were predominantly used [8–19, 30]. However, it has been demonstrated that the expansion is often archial in nature [21]. In that sense, linear distance measurements could produce false differential assessments when the expansion is rotational. Moreover, angular measurements would be more accurate if a true fulcrum can be located. The purpose of this study was to quantify the differential components of MSE expansion by calculating the fulcrum locations and applying a novel angular measurement system.

## Material and methods

This retrospective study was performed at the University of California at Los Angeles (UCLA) with approval by the ethics committee (IRB number 17-000567).

The pre- and post-expansion CBCT images from 39 patients (13 males, 26 females), successively treated with MSE (Biomaterials Korea, Seoul, Korea), with a mean age of 18.2 ± 4.2 years (13.3-27.3), were obtained. All patients were diagnosed with maxillary transverse deficit: thirty-two patients with posterior crossbite (15 bilaterally and 17 unilaterally) and the other seven patients without crossbite. All patients were treated at the orthodontic clinic, UCLA School of Dentistry, under the supervision of one clinician. The orthodontic treatments with bonding of brackets and other appliances were carried out after the completion of MSE expansion and acquisition of post-expansion CBCT. The selection criteria included (1) diagnosis of a transverse maxillary deficiency, (2) cases requiring MSE expansion as part of the overall treatment plan, (3) patient records with CBCT images obtained at 2 times: pretreatment and within 3 weeks after active expansion; (4) absence of any craniofacial irregularity, and (5) no orthodontic treatment precedent.

**Fig. 1:**
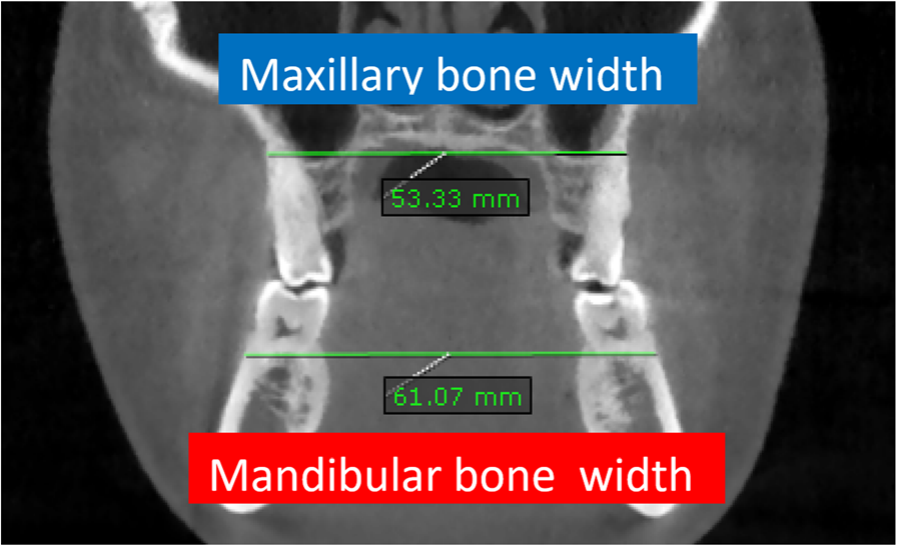
The maxillary bone width is determined by the distance between the right and left bony points at the level of the mesiobuccal root tips of the first molars. Mandibular bone width was defined as the distance between the right and left bony buccal surface at the level of the furcation of first molars. The maxillomandibular bone width discrepancy is obtained by subtracting mandibular bone width (61.07 mm) from the maxillary bone width (53.33) = −7.74 mm

The transverse deficiency was diagnosed by relating the maxillary and mandibular bone width lines in coronal cuts from the initial CBCTs. The maxillary bone width was determined by the distance between the right and left bony points at the level of the mesiobuccal root tips of the upper first molars. Mandibular bone width was defined as the distance between the right and left bony buccal surface at the level of lower first molar furcation. The maxillomandibular bone width discrepancy was obtained by subtracting mandibular bone width from the maxillary bone width. Ideally the maxillary width must be equal or greater than the mandibular width in order to obtain adequate transverse skeletal relationship and allow dental decompensation (Fig. 1).

**Fig. 2:**
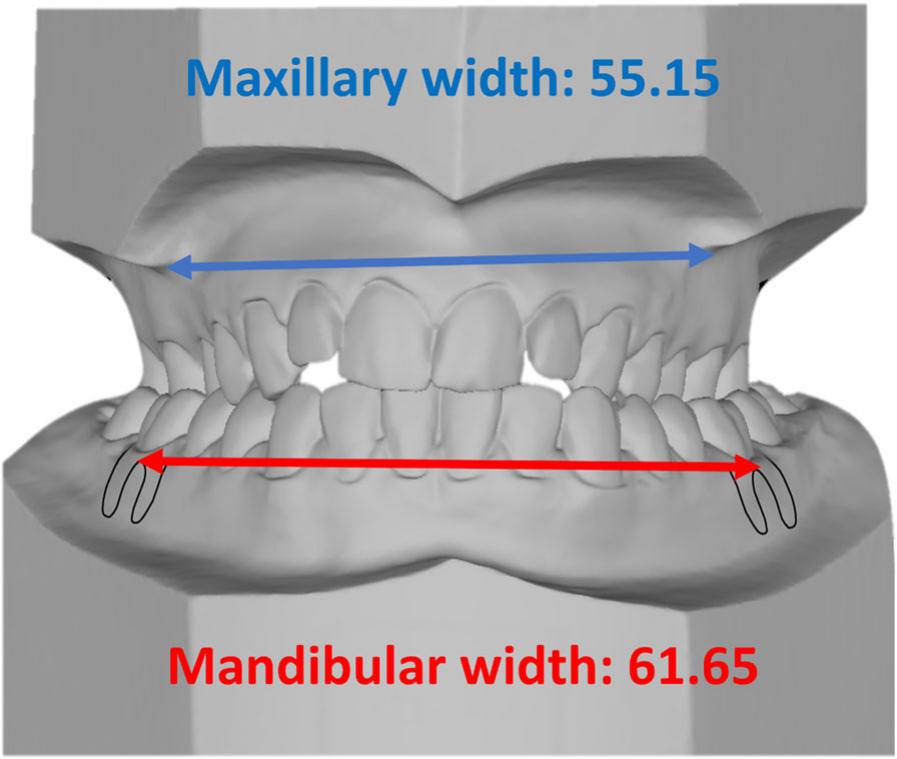
Method used in dental casts to project the extent of maxillary skeletal expansion required. Blue line, maxillary width, and red line, mandibular width measured with a digital caliper; Mandibular width is defined as the distance between the right and left gingiva tissue projected at the level of first mandibular molar’s furcation. In this case, the transverse deficiency in models accounts for 6.5 mm

Furthermore, in clinic and with dental casts, the maxillary width is determined by the distance between the right and left most concave points, lying on the maxillary vestibule above the mesiobuccal cusps of the first molars [31]. Mandibular width is defined as the distance between the right and left buccal surface over the furcation of first molars. The amount of difference among these values projects the extent of maxillary skeletal expansion required (Fig. 2). Taking measurements on study models can be done before and during the expansion in order to assess the bone relationship. With these measurements, the expansion was stopped when an adequate expansion had been achieved. The maxillary width must be wider than the mandibular width in order to produce an optimal occlusion after dental decompensation. The furcation is most likely the center of rotation for mandibular molars during the decompensation. The width between the buccal points over furcation was a projected mandibular width after lower dental uprighting. The maxillary molars are generally flared buccally and the decompensation will involve a constriction of the dental arch. The most concave area of the maxilla is often at the apex of the maxillary molar, and controlled tipping with fulcrum at the apex is required during the decompensation unlike the mandibular molars. Some patients with slight transverse deficiency, but not requiring expansion for normal function (due to well-compensated dentition), were not included in this study.

**Fig. 3:**
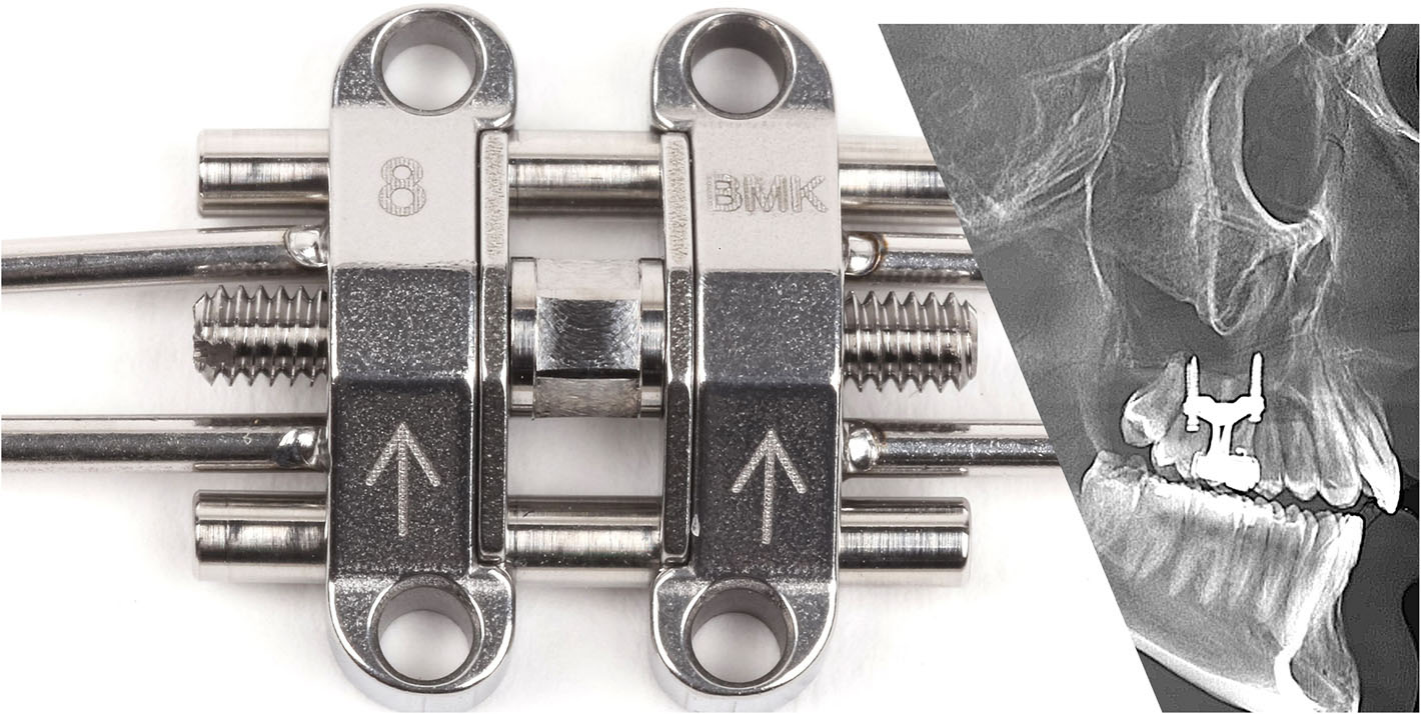
Midfacial skeletal expander device and X-ray showing bicortical engagement of the four microimplants

The MSE device (Fig. 3) has a jackscrew unit (16.15 mm in length, 4.5 mm in width, and 14.15 mm in depth) with four parallel holes (1.8 mm in diameter) for microimplant insertion and two soft supporting arms on each side which are soldered to the molar bands for stabilizing MSE during the expansion. The body of MSE is positioned between the zygomatic buttress bones, usually located lateral to the first molars. Four microimplants (1.8 mm in diameter, 11 mm or 13 mm in length) were inserted through the palatal bone, bi-cortically. The posterior placement and bicortical engagements promote posterior and superior expansion of the maxillary process, which in turn produces the archial expansion described in the previous study [21]. The rate of expansion was 2 activations per day (0.20 mm per turn) until a diastema appeared; then the expansion rate changed to 1 activation per day. The activation was continued until the maxillary skeletal width, was equal or greater than the mandibular width. The MSE was kept in place with no further activation for 6 months to retain the expansion during the bone formation.

In addition to the pre-expansion CBCT scan taken before the expansion, a second CBCT scan was obtained within 3 weeks after completing the expansion. The time interval between the scans was 5 ± 2 months, and this included the time lapsed for administrative procedures, appliance fabrication, delivery, and treatment. In order to assess skeletal outcomes induced purely by MSE, post-expansion scans were obtained before patients received any other orthodontic appliances. The same scanner (5G; NewTom, Verona, Italy) was used for all patients, with an 18 × 16 cm field of view, 14-bit grayscale, and a standard voxel size of 0.3 mm. Configuration of the CBCT included scan time of 18 s (3.6 s emission time), with 110 kV. In order to properly adjust the milliamperes, an automated exposure control system was used to detect the patient’s anatomic density. OnDemand3D (Cybermed, Daejeon, Korea) software was used to superimpose the pre- and post-expansion CBCT images, using the anatomical structures of the entire anterior cranial base [32] by automated processing in matching the voxel grayscale patterns.

**Fig. 4:**
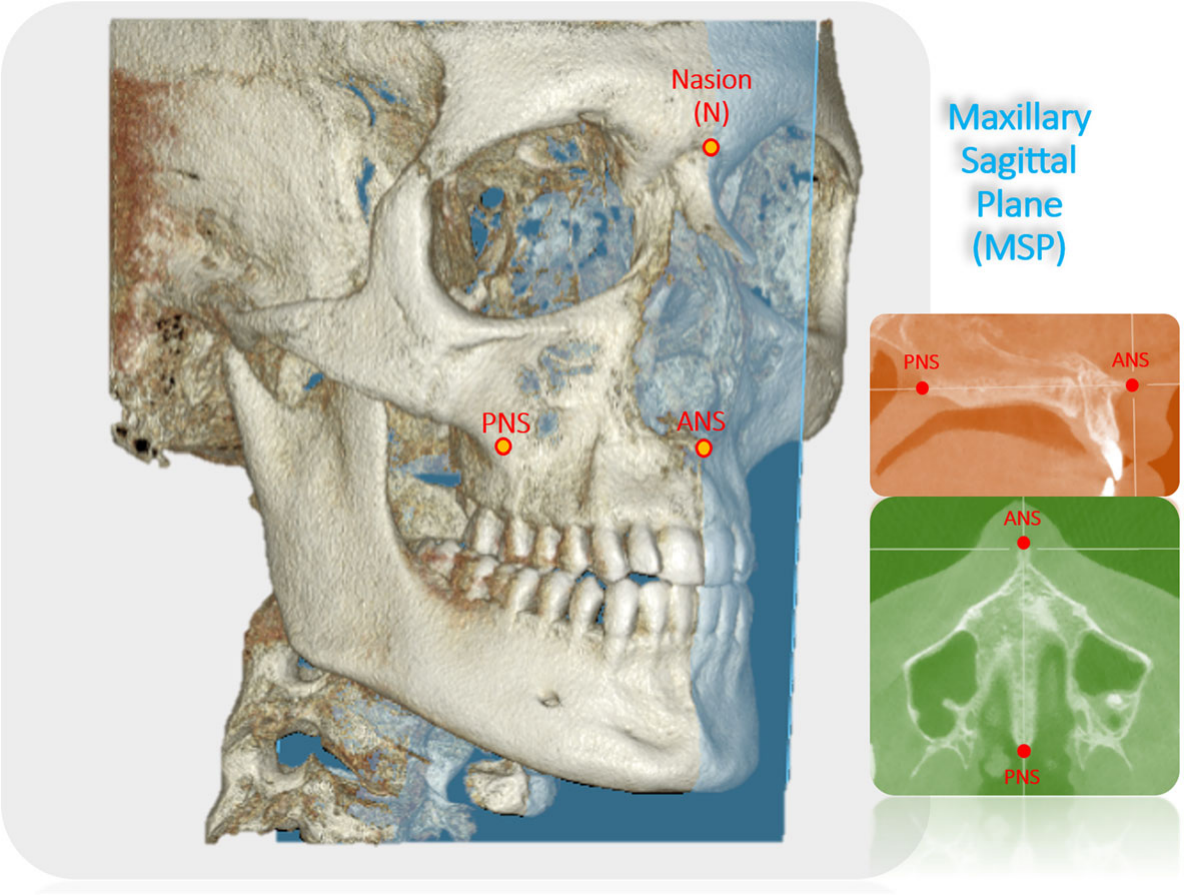
3D reconstruction with the maxillary sagittal plane (MSP) passing through anterior nasal spine (ANS), posterior nasal spine (PNS), and nasion (N) on the pre-expansion CBCT

**Fig. 5:**
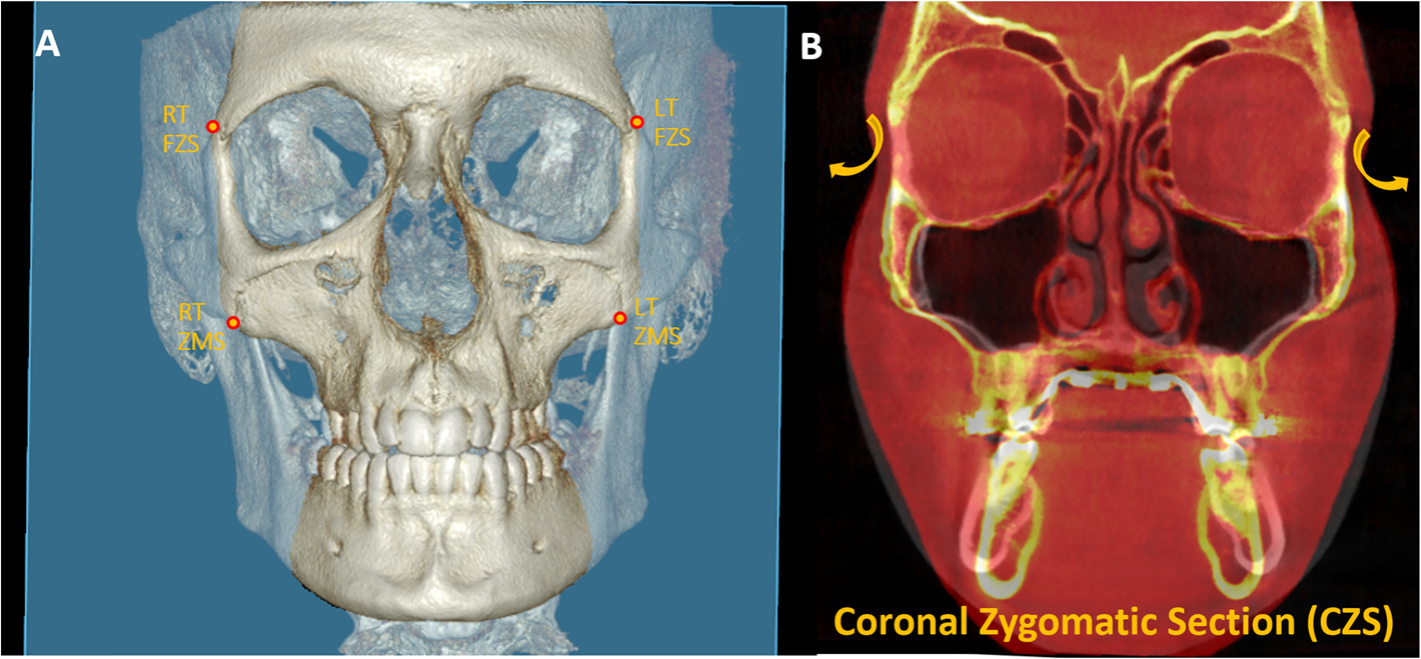
**a** 3D reconstruction with the coronal zygomatic section in blue passing through the right and left frontozygomatic sutures (FZS) and zygomaticomaxillary sutures (ZMS). **b** Pre-treatment and post-treatment superimposed image of an MSE patient in the coronal zygomatic section. The rotational arrows on yellow show the archial movement of the zygomaticomaxillary complex in the coronal plane

**Fig. 6:**
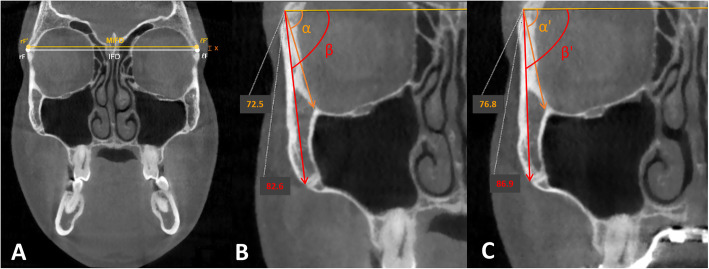
**a** Reference lines to determine rotational fulcrum. Interfrontal distance (IFD) and modified interfrontal distance (MIFD); *x*, distance between IFD and MIFD. According to the Reuleaux technique, at least two corresponding landmarks must show uniform displacement around a single point, to be able to pinpoint a center of rotation. **b** Pre-expansion measurements. **c** Post-expansion measurements. By subtracting the pre-expansion values from the post-expansion values (*α*’−*α* = *β*’−*β*), equals 4.3° of difference

Following the superimposition of pre- and post-expansion CBCT images, the exact fulcrum location of each patient was identified utilizing the following method. The maxillary sagittal plane was identified, passing through the anterior nasal spine, posterior nasal spine, and nasion on the pre-expansion CBCT image (Fig. 4). Then the coronal zygomatic plane (Fig. 5) was selected. This section passes through the uppermost point of the frontozygomatic sutures and the lowermost point of the zygomaticomaxillary sutures. The fulcrum localization was indicated to be near and slightly above the external surface of the frontozygomatic suture because sutures are the weakest points of the midfacial structure during its archial movement after expansion [21]. For this reason, the most external and inferior points of the zygomatic processes of the frontal bones were picked as primary reference landmarks. These two reference points were connected and measured through the interfrontal distance (IFD) on both pre- and post-expansion CBCT images (Fig. 6). If post-expansion measurements were greater than pre-expansion measurements, the exact fulcrum points were located more superiorly. To identify the true fulcrums, a parallel line, slightly above the initial interfrontal line, was moved superiorly upwards until pre- and post-expansion distances were equal. After this was achieved, these newly established lines with no width changes were designated as the modified interfrontal distance (MIFD). The most external points of this MIFD were referred to as the right and left rotational fulcrum respectively. If the initial interfrontal distances (pre- and post-expansion) were the same size, the most external points of this line can be denoted as right and left rotational fulcrum points. In addition, a parallel line slightly below the initial interfrontal line was used to verify that the post-expansion distances were greater than the pre-expansion. In this situation, the modified interfrontal line is the same as the initial interfrontal line (Fig. 6a). To further validate the true center of rotation, two different landmarks were picked in the zygomatic bone and the angular displacement was measured around this proposed fulcrum point (Fig. 6b, c). The angles formed between the modified interfrontal line and the line connecting the proposed fulcrum to two chosen landmarks were measured. The first landmark was the zygomaticomaxillary suture and the second landmark was the junction of the inner zygomatic cortical bone with the floor of the orbit and maxillary sinus in both pre- and post-expansion CBCT. According to the Reuleaux technique [33], at least two corresponding landmarks must show uniform displacement around a single point, to be able to pinpoint a center of rotation. If the changes in pre- and post-expansion degrees were the same for the two angles within each zygoma in all cases, the accuracy of the fulcrum locations was confirmed (Fig. 6b, c).

**Fig. 7:**
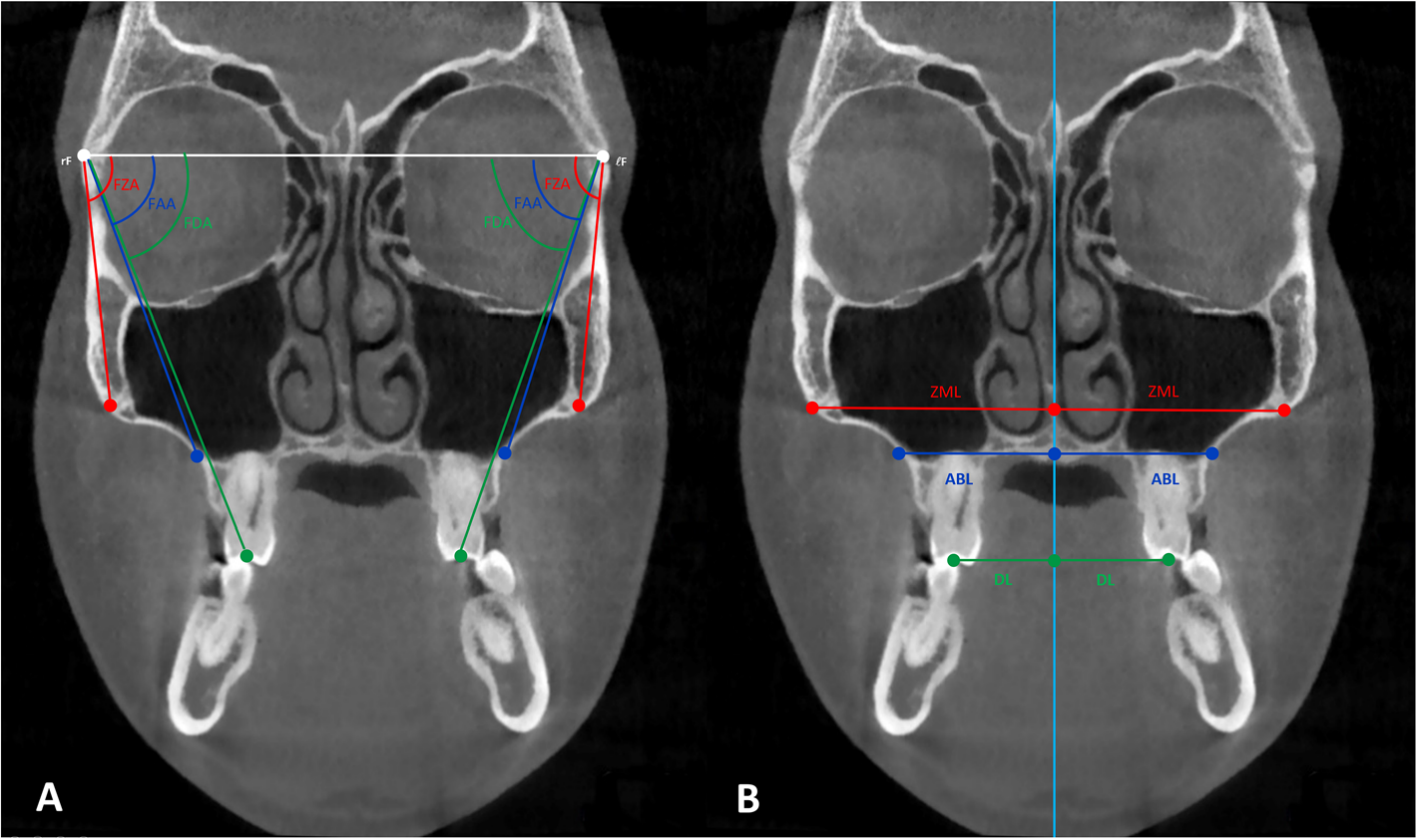
Measurement systems in the coronal zygomatic section. **a** Angular measurement system: frontozygomatic angle (FZA), frontoalveolar angle (FAA), and frontodental angle (FDA); rF, right fulcrum; lF, left fulcrum. **b** Linear measurement system: zygomaticomaxillary line (ZML), alveolar bone line (ABL), and dental line (DL). Light blue line represents the maxillary sagittal plane

After locating the fulcrums and using the same coronal section, two different measurement systems were applied to assess the skeletal, alveolar, and dental components of MSE expansion: traditional linear measurements and a novel angular measurement system (Fig. 7).

From the rotational fulcrum, the following angular measurements were performed: the frontozygomatic angle (FZA) connects the interfrontal line and the line extending from fulcrum to the most external point of the zygomaticomaxillary suture, the frontoalveolar angle (FAA) connects the interfrontal line and the line extending from fulcrum to the alveolar bone surface at the level of distobuccal root tip of the upper first molars, and the frontodental angle (FDA) connects the interfrontal line and the line extending from fulcrum to the occlusal point located at the central groove of the upper first molar (Fig. 7a).

In order to determine the alveolar point for the FAA, a line parallel to the interfrontal line was moved down until it contacted the tip of the root. The alveolar point that intersected the line was selected. These three particular angles were measured on the right and left sides, and the pretreatment value was subtracted from the post-expansion value in order to determine the treatment change for each section. The FZA changes correspond to the zygomaticomaxillary expansion, a true skeletal expansion (FZA changes); the FAA changes correspond to the sum of the skeletal expansion (FZA change) and the alveolar bone bending (FAA changes-FZA changes); and the FDA changes correspond to the sum of the skeletal expansion (FZA changes), alveolar bone bending (FAA changes-FZA changes), and the dental tipping (FDA changes-FAA changes).

In order to contrast with the angular values obtained, a set of traditional linear distance measurements were performed on the same coronal section (Fig. 7b). The zygomaticomaxillary line (ZML), the alveolar bone line (ABL), and the dental line (DL) are perpendicular lines connecting the three landmarks used for the above angular measurements to the intersecting points on the maxillary sagittal plane. Similarly, the changes in the three sections of linear measurements, before and after the MSE treatment, were calculated for right and left sides in order to determine the treatment change. The ZML changes correspond to the zygomaticomaxillary skeletal changes (ZML changes); the ABL changes involve the sum of the skeletal change (ZML change) and the alveolar bone bending (ZML changes-ABL changes); and the DL changes include the sum of the skeletal change (ZML change), alveolar bone bending (ZML changes-ABL changes), and the dental tipping (DL changes-ABL changes).

After determining the fulcrum in all the 39 cases, the three components of the expansion (skeletal expansion, alveolar bone bending, and dental tipping) were assessed by the two measurement systems described above. These results were compared.

### Statistical analysis

Measurements were obtained for the 12 variables (6 pretreatment and 6 post-expansion) on 10 randomly selected patients, by 2 raters, to assess method reliability. Measurements were then repeated after 2 weeks by the same operators, after reorienting the skull according to the reference planes to compute reliability parameters that are the combination of error in the identification of reference planes (maxillary sagittal plane, coronal zygomatic section) and error in landmark localization. The calculated parameters were rater standard deviation, rater coefficient of variation, error standard deviation, error coefficient of variation, and intraclass correlation coefficient (ICC).

For each variable, the pre-expansion value was subtracted from the post-expansion value. The percentages of skeletal expansion (a, the frontozygomatic angle changes), alveolar bone bending (b, the frontoalveolar angle change—the frontozygomatic angle change), and dental tipping (c, the frontodental angle change—the frontoalveolar angle change). A similar calculation was performed for the linear measurements. The mean of the treatment change per each variable was compared with zero, and the *P* value was computed using the Wilcoxon signed-rank test for paired data.

Mean values of the total frontozygomatic, frontoalveolar, and frontodental angle changes were compared using the Kruskal-Wallis test.

## Results

The mean maxillomandibular bone width discrepancy, assessed in CBCT, accounted for 5.2 (± 3.4) mm. The average amount of activation of the MSE expansion jackscrew was 8.7 ± 1.2 mm. The duration of maxillary expansion ranged from 15 to 36 days. From the 39 patients, 20 subjects have the rotational fulcrum of the zygomaticomaxillary complex at the most distant points of the interfrontal distance (101.6 ± 4.7 mm) and 19 subjects at the most distant points of the modified interfrontal distance (98.9 ± 5.7 mm). The modified interfrontal distance was found to be at 0.6 ± 0.29 mm above the interfrontal distance (range 0.19-1.01 mm). Pre-expansion and post-expansion linear and angular measurements are presented in Tables 1 and 2.

**Table 1:** Skeletal, alveolar bone, and dental linear measurements

Linear measurements		Before expansion		After expansion		Treatment change		
	Unit	Mean	SD	Mean	SD	Mean	SD	*P* value
Skeletal linear measurements								
Right zygomaticomaxillary line	mm	44.46	2.54	46.77	2.65	2.31	1.02	< 0.0001*
Left zygomaticomaxillary line	mm	44.30	2.49	46.67	2.62	2.37	1.18	< 0.0001*
Alveolar bone linear measurements								
Right alveolar bone line	mm	29.07	2.32	32.01	2.53	2.93	1.16	< 0.0001*
Left alveolar bone line	mm	29.38	2.23	32.45	2.33	3.06	1.47	< 0.0001*
Dental linear measurements								
Right dental line	mm	22.44	2.69	26.28	2.66	3.84	1.65	< 0.0001*
Left dental line	mm	23.12	2.42	27.30	2.32	4.17	1.86	< 0.0001*

*SD* standard deviation

*P < 0.01

**Table 2:** Skeletal, alveolar bone, and dental angular measurements

Angular measurements		Before expansion		After expansion		Treatment change		
	Unit	Mean	SD	Mean	SD	Mean	SD	*P* value
Skeletal angular measurements								
Right frontozygomatic angle	°	83.47	3.60	86.29	3.47	2.82	1.26	< 0.0001*
Left frontozygomatic angle	°	83.25	3.49	86.19	3.88	2.93	1.49	< 0.0001*
Alveolar bone angular measurements								
Right frontoalveolar angle	°	70.20	2.51	73.03	2.38	2.83	1.27	< 0.0001*
Left frontoalveolar angle	°	70.44	2.85	73.39	3.27	2.94	1.48	< 0.0001*
Dental angular measurements								
Right frontodental angle	°	69.37	2.32	72.29	2.09	2.92	1.29	< 0.0001*
Left frontodental angle	°	69.92	2.19	72.99	2.54	3.07	1.48	< 0.0001*

*SD* standard deviation

*P < 0.01

The treatment change for the 39 cases with linear measurements at the zygomaticomaxillary level was 2.31 (± 1.02) and 2.37 (± 1.18) mm (right and left sides) at the alveolar bone level was 0.62 (± 0.44) and 0.69 (± 0.46) mm (right and left sides), and at the dental level was 0.91 (± 0.73) and 1.11 (± 0.6) mm (right and left sides). These values suggest 60.16% and 56.83% (right and left sides) skeletal expansion, 16.15% and 16.55% (right and left sides) alveolar bone bending, and 23.69% and 26.62% (right and left sides) dental tipping. In contrast, the treatment change with angular measurements at the zygomaticomaxillary level was 2.82° (± 1.26), and 2.93° (± 1.49) (right and left sides) at the alveolar bone level was 0.01° (± 0.03) (both right and left sides), and at the dental level was 0.09° (± 0.17) and 0.13° (± 0.12) (right and left sides). These values represent 96.58% and 95.44% of skeletal expansion for the right and left sides, 0.34% and 0.33% alveolar bone bending for right and left sides, and dental tipping of 3.08% and 4.23% for the right and left sides respectively (Table 3).

**Table 3:** Linear and angular treatment change measurements for each component

				Skeletal expansion	Alveolar bone bending	Dental tipping
	Right linear measurements					
Unit	Δa	Δb	Δc	Δa	Δb—Δa	Δc—Δb
mm	2.31	2.93	3.84	2.31	0.62	0.91
%				60.16	16.15	23.69
	Right angular measurements					
	Δa	Δb	Δc	Δa	Δb—Δa	Δc—Δb
°	2.82	2.83	2.92	2.82	0.01	0.09
%				96.58	0.34	3.08
	Left linear measurements					
	Δa	Δb	Δc	Δa	Δb—Δa	Δc—Δb
mm	2.37	3.06	4.17	2.37	0.69	1.11
%				56.83	16.55	26.62
	Left angular measurements					
	Δa	Δb	Δc	Δa	Δb—Δa	Δc—Δb
°	2.93	2.94	3.07	2.93	0.01	0.13
%				95.44	0.33	4.23

*Δa* skeletal expansion, *Δb* skeletal expansion + alveolar bone bending, *Δc* skeletal expansion + alveolar bone bending + dental tipping

There was no significant difference between the mean values of the total frontozygomatic, frontoalveolar, and frontodental treatment change angles (*P* = 0.748) (Table 4).

**Table 4:** Mean values of the total frontozygomatic, frontoalveolar, and frontodental angles

	Treatment change at ZM point		Treatment change at AB point		Treatment change at D point		
Unit	Mean	SD	Mean	SD	Mean	SD	*P* value
°	2.87	1.37	2.88	1.37	2.99	1.38	0.748

*ZM* zygomaticomaxillary; *AB* alveolar bone; *D* dental; *SD* standard deviation

For the considered parameters, the rater coefficient of variation was 1.36% or less, and the error coefficient of variation was 1.75% or less, showing that measurements were highly reliable.

## Discussion

It is desirable to design an appliance that produces skeletal maxillary expansion with minimal dentoalveolar consequences. In designing an expansion device, the main considerations should be maximizing the orthopedic expansion of the midcranial structure without significant dentoalveolar changes, by applying the force directly against the resisting structures. Maintenance of surrounding tissue integrity and stability, while achieving desired dimensional changes in an efficient and consistent manner, is desired. The MSE is specifically designed to achieve a skeletal midfacial expansion by applying the expansion force directly against the midpalatal suture and zygomatic buttress bones in order to minimize the negative dentoalveolar side-effects. This resulted in archial rotations of midcranial structures with the fulcrums located near the frontozygomatic sutures [21]. With this information, a method of determining the precise fulcrum locations and a novel measurement system utilizing the angular changes from these fulcrums were developed. In order to apply this measurement system, it is vitally important to first determine the correct fulcrum location for each individual patient. Furthermore, the fulcrum location used in the current study can only be applied to MSE patients because the fulcrum location most likely will differ with each appliance design and activation protocol.

The treatment timing is another factor to consider when maxillary expansion is performed. The resistance against skeletal separation increases beyond the pubertal growth period, and a significant sutural separation cannot be anticipated with tooth-borne expanders, causing more dentoalveolar changes. It has been believed that, by young adulthood, fusion of the sutures virtually eliminates the potential for sutural separation without surgical assistance [34]. Even in growing patients, heavy forces of RME produce an increased buccal inclination of anchored teeth at the end of the active phase, regardless of the type of expanders [3, 4, 10, 35–38] or of the rate of activation [8]. Also in pre-pubertal patients, it has been demonstrated that alveolar structures splayed buccally and carried the teeth with them [3, 37], and that a 6-month period is necessary to allow recovery of the alveolar plate [39]. This adverse effect was observed in both adolescents and adult subjects when tooth-borne devices were used. However, the undesirable consequences such as dentoalveolar tipping, fenestration, dehiscence, and gingival recession [5] were more common and critical with post-pubertal patients. Furthermore, the dental tipping gradually relapsed after the active expansion phase even in growing patients [6, 8], indicating the importance of avoiding the dentoalveolar changes during the expansion.

**Fig. 8:**
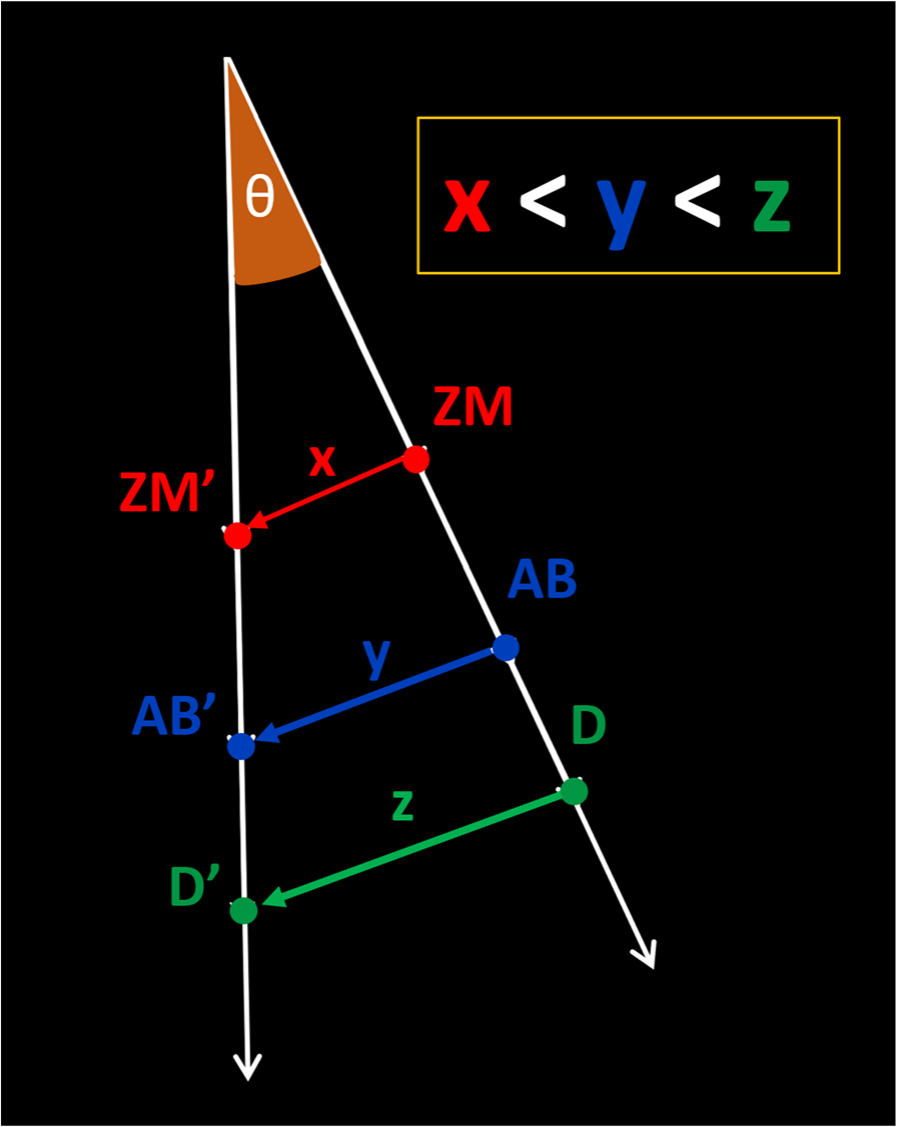
Diagram displaying the inaccuracy of using linear measurements in order to assess rotational pattern movement. For the same angle *θ*, points closer to the fulcrum experience a shorter linear displacement than points farther from the fulcrum. ZM, pre-expansion zygomaticomaxillary point; ZM’, post-expansion zygomaticomaxillary point; AB, pre-expansion alveolar bone point; AB’, post-expansion alveolar bone point; D, pre-expansion dental point; D’, post-expansion dental point; *x*, linear skeletal distance; *y*, linear alveolar bone distance; *z*, linear dental distance

With the introduction of microimplants to the RPE appliances, a new non-surgical alternative treatment for maxillary deficiency patient has been established. Since many of these appliances are bone-born in nature, the MARPE should not affect dentoalveolar structures. Nonetheless, expression of a pure skeletal expansion has been negated in several articles [9–19, 40], and they present various amount/percentage of alveolar bone bending and dental tipping. There are two problems associated with these studies. These studies employed linear measurements to differentiate three components of the expansion. This approach has an inherent error shown in Fig. 8. When the expansion is archial in nature, the movement of a structure further away from the fulcrum point will be displaced further when linear measurements are used. Although linear measurements are not accurate, in our daily orthodontic diagnosis and practice, transverse dimensions are still practically measured on the horizontal plane. But this can lead to an error in assessing the differentiation of the three components (skeletal, alveolar, and dental) of expansion since the dental and alveolar components are further away from the fulcrum point. In the current study, the angular measurements were employed in order to overcome the above problems. The proposed angular measurements reflect the true differential movements of the three components (skeletal, alveolar, and dental movements). This approach is possible only if the true fulcrums have been defined. The conventional linear measurements were also applied to all patients, in order to assess the differences between the two measurement systems.

To assess the MSE outcomes, the fulcrum position of the zygomaticomaxillary complex on the coronal plane was determined based on the Cantarella study [21]. When the angular measurements were used from these fulcrum points, the MSE produced almost pure skeletal expansion (2.82° = 96.58% R; 2.93° = 95.44% L) negligible alveolar bone bending (0.01° = 0.34% R; 0.01° = 0.33% L) and with a slight dental tipping (0.09° = 3.08% R; 0.13° = 4.23% L), in contrast to other MARPE studies. There was no significant difference between the total mean values of the frontozygomatic, frontoalveolar, and frontodental treatment change angles (*P* = 0.748). The angular changes (counting both right and left values together) indicating the alveolar bone bending and dental tipping were not statistically different than the angular change indicating the skeletal changes (Table 4), which illustrates that the MSE expansion was mostly skeletal with entire midcranial structures rotating from the fulcrum points.

**Fig. 9:**
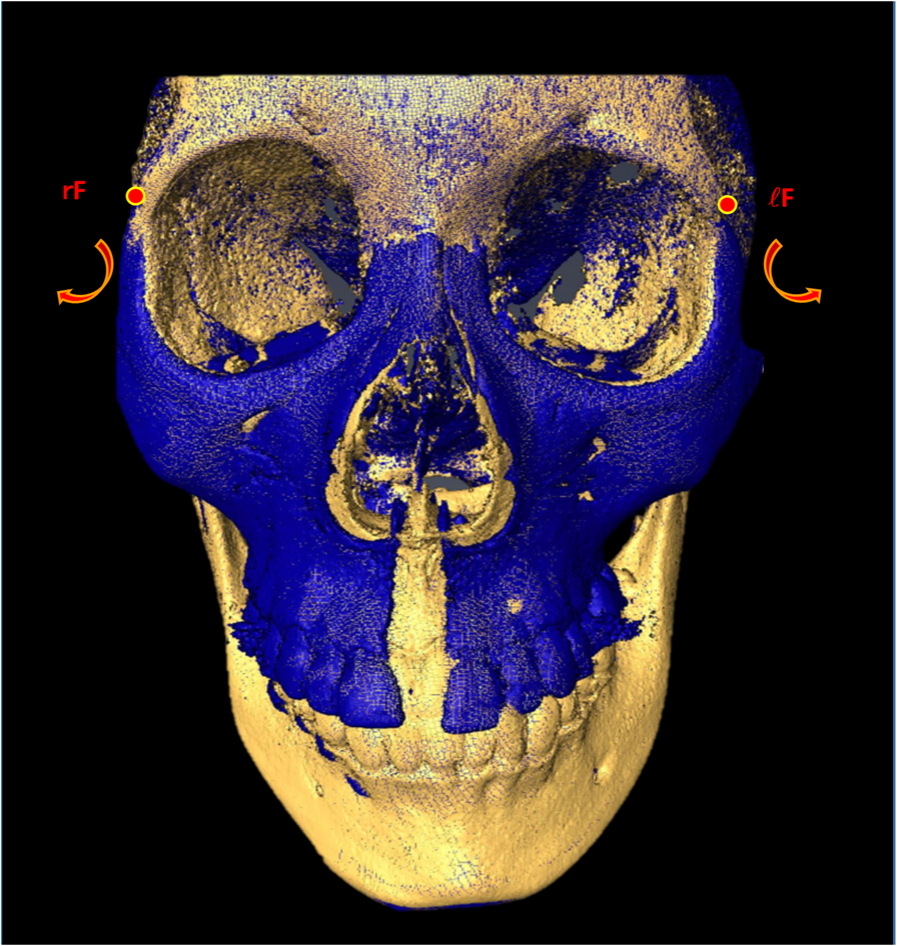
Superimposed 3D model of an MSE patient displaying the rotational pattern of the zygomaticomaxillary complex. Yellow, pre-expansion; blue, post-expansion; rF, right fulcrum; ℓF, left fulcrum. Structures medial and above the fulcrum are stable. Red arrows show the outward and downward direction of the expansion on the coronal view

The results were obtained by MSE treatment for late adolescents and adults. The principal features related to the above results were the bicortical engagement [20] of the four microimplants (11 to 13 mm length) placed immediately next to the midpalatal suture and the MSE location in between the zygomatic buttress bones. The bicortical engagements of the microimplants promote the expansion force to reach the superior aspect of the maxillary complex [20]. The anatomical location of MSE produced a force vector in line with the zygomatic bone [28] and produced the midcranial movement. These two factors together produced the rotation of the midcranial structure at a high fulcrum position (Fig. 9). Clearly, the MSE produced a pure rotation of midfacial structures with negligible alveolar bone bending or dental tipping.

The linear measurement system was used on the same data set, in order to assess the inherent error built into this system. The results from the linear measurement were quite different than those from the angular measurements: the skeletal expansion (2.31 mm = 60.16% R; 2.37 mm = 56.83% L), negligible alveolar bone bending (0.62 mm = 16.15% R; 0.69 mm = 16.55% L), and with a slight dental tipping (0.91 mm = 23.69% R; 1.11 mm = 26.62% L). When dealing with a rotational movement, this type of measurement system has severe shortcomings because it does not account for the differences in radius of each variable. The structure further away from the fulcrum has a longer radius, and the linear length of the movement is longer, producing a false differential movement. Other studies related to the archial movement of the structures could have suffered the same consequences if this type of linear measurements was employed. However, the angular measurements from arbitrary points cannot produce accurate readings either. Without an accurate fulcrum location, even the angular measurements can produce false assessments.

Lin et al. [9] had a comparison study between tooth-borne and bone-borne MARPE on late adolescents. The MARPE used in this study included 4 microimplants embedded in two acrylic shelves supporting the jackscrew. All implants were positioned close to the dentition, inferiorly from the midpalatal suture, but the appliance did not contact the dentition. Angular measurements were employed to assess alveolar bone bending and dental tipping using an arbitrary palatal plane. They found a significant alveolar bone bending and dental tipping even with this bone-borne expander treatment. Because of the force applied to the dentoalveolar region by this appliance, the dentoalveolar changes may have been possible; however, it is difficult to accept that dental movement can occur when the expander did not have any physical contact with the dentition. This implies that angular measurements from arbitrary points cannot accurately assess the true impact of an appliance. Similarly, many have used arbitrary reference lines and points to assess the results of expansion, without considering the fulcrum position.

The actual dental tipping and alveolar bone bending may be much less than the reported values in many instances when the movement was rotational in nature. The challenge is locating the true fulcrum for each appliance in question. Further study will be necessary in order to determine the best way to identify the fulcrum for various appliance design. Once the fulcrum is located, the angular measurements similar to the system proposed in this study can be useful in accurately determining the effect of expanders. Comparative studies with conventional tooth-borne appliances, other bone-borne expanders and surgically assisted rapid palatal expansion (SARPE), using the novel method presented here will be useful in understanding the real differences between these groups of expansion modalities.

The tooth-borne, pure bone-borne, tooth-and-bone-borne, bone-and-tissue-borne all behave differently. Furthermore, each appliance within the same type can exhibit completely different expansion pattern. Moreover, the position of the expander can alter the fulcrum position and expansion pattern. It is not possible to understand the expansion configuration for each appliance without identifying the exact fulcrum locations. Once the fulcrum has been established, the angular measurements can be taken. We could expect more dental components from tooth-borne and tooth-and-bone-borne appliances. However, the alveolar bone bending probably is more related to the expansion force delivery relative to the resisting structures, which is defined by the position of bone and tooth anchors. More inferior they are, generally, will cause more alveolar bone bending.

Limitations of this study are related to its retrospective nature, and the lack of a control group due to ethical issues. Although the values obtained from the current study are applied to the MSE, the system presented in this article could not be used for other types of expanders. Every expander has a different design an activation protocol. These factors may vary the position of the fulcrum.

## Conclusions

1. MSE produced almost pure skeletal rotational movement of the midcranial structures.
2. Alveolar bone bending and dental tipping were not statistically significant with MSE.
3. The angular measurement system from fulcrums provided much different results than the linear measurement system. The conventional linear measurements can falsely exaggerate the alveolar and dental components of MSE treatment.
4. To correctly differentiate the expansion pattern of the rotating zygomaticomaxillary complex, a localization of the fulcrum should be the first step, then the angular measurements should be performed.
5. Fulcrum position may vary depending on the design of the expander and the activation protocol, and a true fulcrum for each appliance should be identified for the proposed angular measurement system.

## Data Availability

Data of the present study will not be shared because the same data and materials will be used in further publications where the analysis of different midface bones and sutures will be presented.

## Abbreviations

CBCT: Cone-beam computed tomography
MSE: Midfacial skeletal expander
MARPE: Miniscrew-assisted rapid palatal expansion
RPE: Rapid palatal expansion
SARPE: Surgically assisted rapid palatal expansion
IRB: Institutional Review Board
MSP: Maxillary sagittal plane
ANS: Anterior nasal spine
PNS: Posterior nasal spine
N: Nasion
CZS: Coronal zygomatic section
FZS: Frontozygomatic suture
ZMS: Zygomaticomaxillary suture
IFD: Interfrontal distance
MIFD: Modified interfrontal distance
FZA: Frontozygomatic angle
FAA: Frontoalveolar angle
FDA: Frontodental angle
ZML: Zygomaticomaxillary line
ABL: Alveolar bone line
DL: Dental line
R: Right
L: Left
SD: Standard deviation
ICC: Intra-class correlation coefficient
